# The Interrelation Among Allometry, Phylogeny, and Diet in the Molar Cervix

**DOI:** 10.1002/ajpa.70321

**Published:** 2026-07-16

**Authors:** Zana R. Sims

**Affiliations:** ^1^ Department of Ecology, Evolution & Behavior University of Minnesota Saint Paul Minnesota USA

**Keywords:** allometry, dentition, diet, morphometrics, phylogeny

## Abstract

**Objectives:**

Root surface morphology has been used to examine functional signals related to diet and taxonomic affinities in primates with varying degrees of success. The aim of this study is to determine if the use of the functionally significant root cervix can enhance our understanding of these relationships in catarrhines.

**Materials and Methods:**

Two‐dimensional (2D) semilandmark points were collected using cross sections taken from the cervix of all three mandibular molar positions in a sample of extant catarrhines (*n* = 68). Multivariate regression was used to examine the influence of size on Procrustes aligned data. Standard and phylogenetic principal component analyses were performed for each molar position. Phylogenetic generalized least squares (PGLS) were used to correct for nonindependence.

**Results:**

Allometric effects were statistically significant (*p* < 0.05) but weak for each position (*M*_1_ = 0.096, *M*_2_ = 0.064, *M*_3_ = 0.062). Phylogenetic signal, multivariate *K*, was significant for each molar but less than expected under Brownian motion (*K*_mult_ < 1). PGLS removed all functional signal related to dietary ecology at *M*_1_ for phylogenetically aligned PCA (PACA) and phylogenetic PCA (pPCA) components. These signals were diminished but not eliminated at *M*_2_ (PACA) and *M*_3_ (pPCA).

**Conclusions:**

The influence of size on the morphology of the root cervix is limited and does not impact functional signals related to either phylogeny or diet. Observed morphological variation of the root cervix is driven in part by both phylogeny and diet. However, these variables share a strong relationship, and more work is needed to disentangle the two.

## 1 Introduction

Dental morphology is central to the assessment of dietary adaptations, taxonomic affinities, and evolutionary relationships in extant and fossil primates (Kay 1975; Kay and Ungar 1997; M'Kirera and Ungar 2003; Suwa et al. 2007; McNulty et al. 2015; Rasmussen et al. 2019). Through the mathematical analysis of shape we can enhance our understanding of the presence of subtle differences in crown morphology that facilitate ecological divisions, such as diet, between taxa and even among sympatric species (e.g., Ledogar et al. 2013; Winchester et al. 2014; Berthaume and Schroer 2017). The crown feature or features selected study often have functional relevance such as cusps, crests, or basins (e.g., Hlusko 2002; Singleton et al. 2011; Schwartz et al. 2020). Explorations of the form and function of the dental root system came as a natural extension of work on the dental crown (e.g., Kovacs 1979; Wood et al. 1988; Spencer 2003; Kupczik and Hublin 2010; Emonet et al. 2012; Le Cabec et al. 2013; Deutsch et al. 2022). Much of this work, however, has been focused on the use of linear measurements and surface areas; alternative methodologies for the study of root shape, such as landmarks, are underutilized (but see Moore et al. 2013; Kupczik et al. 2018).

The application of landmark‐based geometric morphometric (GM) methods to teeth has resulted in a wealth of new information for both living and extinct primates (e.g., Gamarra et al. 2016; St. Clair and Boyer 2016). Advancements in morphometrics such as the use of sliding semilandmark methods on areas that lack the features necessary for traditional landmark analysis (e.g., Bookstein 1997; Perez et al. 2006; Gunz and Mitteroecker 2013) enabled the quantification of regions like dental basins or enamel‐dentin junctions with fewer homologous landmarks (e.g., Skinner et al. 2009). Research on root morphology has lagged behind the crown and the application of methods like sliding semilandmark analyses are limited. As a result, important signals related to diet, function, or a combination thereof may have been overlooked.

### 1.1 Primate Root Form

Early studies of root morphology used linear measurements to demonstrate that roots could be used to differentiate among taxonomic groups (e.g., Abbott 1984; Wood et al. 1988) For example, Abbott (1984) found that molar root metrics (such as length, angulation, mesiodistal neck diameter, and bifurcation height) were statistically different across great apes and hominins. Abbott suggested that variation in root form provided key insight into evolutionary trends in later hominins toward the simplification or increasing complexity in premolars (Abbott 1984). These foundational studies demonstrated that key information could be obtained by using the roots to reconstruct evolutionary relationships and examine morphological trends.

Functional signals have also been detected using postcanine roots across a variety of primate and nonprimate mammals using measures of surface area (e.g., Spencer 2003; Kupczik 2003; Kupczik et al. 2018; Deutsch et al. 2022). A study by Deutsch et al. (2022) found that the relationship between molar root surface area and diet could be influenced by choice in scaling variable across primates. When scaled by cranial geometric mean, derived from nine measurements including cranial length, breadth, and height as well as mandibular length and corpus height, surface area increased in species with predominantly folivorous diets relative to species with softer diets like frugivory in their sample; however, when root surface area was scaled to body mass, no such signal was discernable suggesting that surface area is more reflective of the size of the animal (Deutsch et al. 2022). This was in contrast to the findings of Spencer (2003) and Kupczik (2003), who found significant associations between larger root surface areas and animals whose diets included the consumption of challenging food items and/or required repetitive loading. The conflicting results of these studies suggest that our understanding of the form‐function relationship in tooth roots remains in its infancy, and research that validates or challenges current interpretations will enhance our knowledge of the root complex.

Several studies have advanced the technical aspects of measuring root form while greatly expanding our understanding of the root system (Kupczik and Dean 2008; Kupczik et al. 2009, 2018, 2019; Cobb and Baverstock 2009; Kupczik and Hublin 2010; Emonet et al. 2012; Le Cabec et al. 2013; Deutsch et al. 2022). For example, Cobb and Baverstock (2009) used 2D fixed landmarks from radiographs of the lower toothrow of *Pan troglodytes* to demonstrate that postcanine root length strongly covaried with facial height but not facial length or sex. The authors put forth three hypotheses to explain this relationship, one of which was a plastic response to loading where roots develop to resist forces after they have entered occlusion but not yet terminated growth (Cobb and Baverstock 2009). Additionally, they suggested the possibility that the stability produced by root length may act to limit the forces that can be generated at each tooth position and lastly, that root length may reflect the variable distances that each erupting tooth must cover to reach occlusion (Cobb and Baverstock 2009). This research emphasizes that a variety of methodological approaches can be beneficial for generating new hypotheses and exploring potential functional signals in root morphology.

The research reviewed above provides a substantial foundation for understanding the potential information that can be obtained from the application of morphology‐based analyses to the primate root system. To date, these studies offer limited and sometimes conflicting evidence regarding the connection between root form, function, and diet. The literature on primate tooth roots is largely concerned with the quantification of external morphological variation, whereas the exploration of the cervix is more limited. As a result, there may be more information that can be obtained through additional investigation. The present study seeks to understand the relationship between the root system, dietary ecology, and phylogeny. To do so, 2D sliding semilandmarks were used to quantify the shape of the root at the level of cervix. The approach of using a cross‐section will fill the gap in our knowledge regarding the morphological variation of molar roots by quantifying how changes in the internal shape vary in relation to the external shape and will test for both dietary and phylogenetic signals to expand our understanding of the impact of these variables on the root system. Because this study sample overlaps with the sample from Sims (2025) it can be used to assess whether landmark analysis reveals additional patterns or signals not detected by cross‐sectional geometry alone. This research describes how the morphology of dentin at the root cervix varies across extant catarrhines and links these differences in form to distinct dietary categories or phylogenetic histories.

## 2 Materials and Methods

### 2.1 Sample

A sample of 191 catarrhine primate molars (*M*_1_ = 64, *M*_2_ = 66, *M*_3_ = 61) were collected from micro‐CT scans with representatives of both major extant superfamilies, Hominoidea and Cercopithecoidea. All molars in the sample were permanent adult teeth identified by full crown eruption above the alveolus and completed root apices (Schultz 1935; Dean et al. 2022). No attempt was made to further partition the sample into age‐based categories. Taxa were chosen to sample broadly from the tree and to obtain a variety of dietary profiles. As outlined in greater detail in the previous study, each dietary category assignment was made using an arbitrary cut‐off of 60% or greater for the consumption of a single food item (e.g., foliage). When consumption percentages for two food items approached 50%, that primate was designated as a “mixed” feeder (e.g., fruit and foliage). Finally, if consumption percentages were variable with no single item reaching the 60% cut‐off, then that primate was designated as an omnivore (Sims 2025). While these primate dietary categories include overlapping food items (e.g., Conklin‐Brittain et al. 2001) as well as food material properties (see Coiner‐Collier et al. 2016), they provide an entry point for the exploration of dietary signal and a consistent method for categorization as long as the method for designation is explicit. Table 1 provides the dietary classifications as well as sample sizes for each genus.

**TABLE 1:** List of primate genera and sample sizes for each tooth position in the study.

Genus	*M*_1_	*M*_2_	*M*_3_	Diet
*Cercocebus*	5	5	4	Hard object frugivore
*Colobus*	5	6	6	Folivore
*Erythrocebus*	3	4	4	Omnivore
*Gorilla*	9	9	8	Mixed folivore
*Hylobates*	9	9	9	Soft object frugivore
*Miopithecus*	5	5	5	Omnivore
*Pan*	8	7	9	Soft object frugivore
*Papio*	5	6	4	Omnivore
*Pongo*	9	9	9	Hard object Frugivore
*Symphalangus*	3	3	1	Mixed Folivore
*Theropithecus*	3	3	2	Folivore

*Note:* References for the assignment of dietary categories can be found in Sims (2025).

### 2.2 Image Processing and Landmarks

**FIGURE 1:**
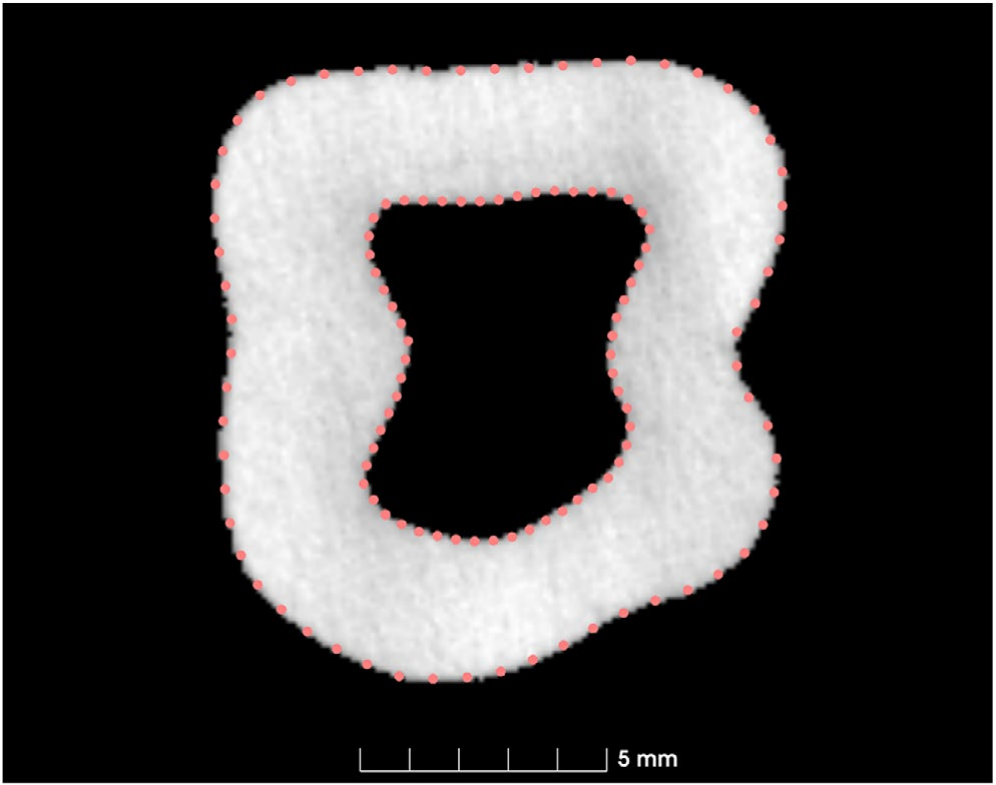
Position of semilandmark curves for the *M*_2_ of specimen MCZ 37516. Scale bar = 5 mm.

Image stacks were imported into Dragonfly ORS version 2022.1 (Dragonfly ORS 2022) and processed using the methods previously outlined in Sims (2025). Individual cross‐section files were loaded into 3D Slicer v. 5.2.2 and the image resolution was adjusted using voxel size. To collect landmark points, the built‐in closed curve tool from the markups module was used. Two curves were manually placed on each cross‐section to capture the shape of the root cervix. The first curve was applied to outline the internal pulp cavity, capturing the contour of any secondary dentin deposition, and the second curve was applied to the external cervical margin. Both curves were resampled to generate 60 evenly spaced points along each curve (Figure 1).

### 2.3 Analysis

Landmark coordinates, curve definitions, and a classifier data file were imported into RStudio v. 4.3.1 (R Core Team 2021). The packages Geomorph (Baken et al. 2021; Adams et al. 2022), Phytools (Revell 2012), Dplyr (Wickham et al. 2023), RRPP (Collyer and Adams 2018, 2023), Geiger (Pennell et al. 2014), Ape (Paradis and Schliep 2019), Pracma (Borchers 2022), and Ggplot2 (Wickham 2016) were used for analysis and visualization. The semilandmark coordinates and corresponding classifier data were subset by tooth position (i.e., *M*_1_, *M*_2_, and *M*_3_). Using the function gpagen, semi‐landmarks for all three teeth combined and then each tooth locus were allowed to slide along tangent vectors using the criterion to minimize Procrustes distances between semi‐landmark positions (Bookstein 1997; Perez et al. 2006). This process was performed concurrently with a generalized Procrustes analysis (GPA) to remove the effects of location, orientation, and scale by moving all observations to the origin, optimizing the alignment of corresponding points across the sample through iterative rotation, and scaling all observations to unit centroid size (Rohlf 1996; Bookstein 1997; Gunz and Mitteroecker 2013). Once a stable landmark configuration was obtained, the sliding process ceased, and the aligned landmark and semi‐landmark coordinates were used as shape variables in subsequent analyses.

The combined Procrustes coordinates were used in a Procrustes analysis of variance (ANOVA) with permutation to test for statistically significant differences among the tooth positions using the function “procD.lm.” with 10,000 permutations. Pairwise comparisons using the function “pairwise” were performed to test the distances between group means and to understand the differences among tooth positions.

To test for the effects of size on the sample, a multivariate regression of the Procrustes coordinates against log_10_‐transformed centroid size was performed using the function “procD.lm,” with 10,000 permutations computed to test for significance. For statistically significant models (*p <* 0.05), residuals were used in an ANOVA with randomized residuals in a permutation procedure (RRPP) to test for differences between the dietary categories while controlling for size with the function “lm.rrpp.” Pairwise comparisons were performed for significant models as above.

**FIGURE 2:**
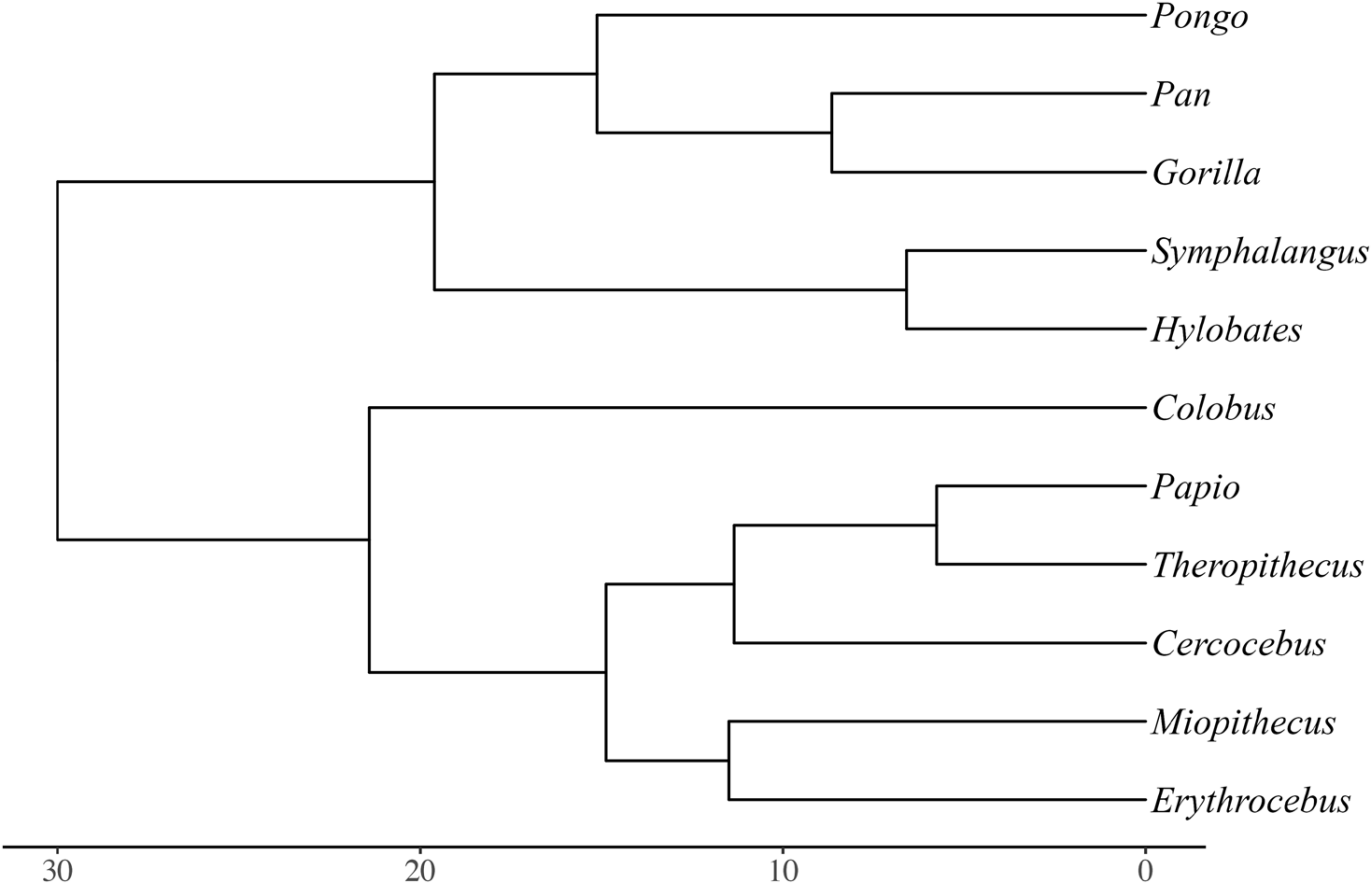
Time‐calibrated consensus tree of the genera included in this study. Scale in millions of years.

To assess the potential impact of phylogenetic relatedness on the morphological patterns observed in these taxa, a time‐calibrated consensus tree of the extant taxa in the sample was downloaded from 10 k Trees (Arnold et al. 2010) (Figure 2). The tree was used to test the Procrustes coordinates and centroid sizes for the strength of the phylogenetic signal on group means using the function “physignal” to obtain a *K*‐statistic for univariate data (i.e., centroid size) using a standard Kappa statistic (*K*) and for multivariate data (*K*_mult_) with 10,000 permutations. Phylogenetic signal refers to the tendency for species that share close evolutionary relationships to demonstrate similar morphologies or values when traits are measured (Blomberg et al. 2003; Adams 2014). Using a Brownian motion model of evolution, *K*_mult_ has an expected value of 1, whereas data that return values greater or less than 1 have stronger or lesser phylogenetic signal, respectively, than the expectation under the model (Adams 2014). Permutation tests are performed through a randomization procedure using the phylogenetic tree tips (genus means of Procrustes coordinates) to provide a statistical evaluation of whether the observed value of *K*_mult_ is significantly different from the randomized data where the value of *K*_mult_ would be zero (Blomberg et al. 2003; Adams 2014).

A principal component analysis (PCA) was performed on the Procrustes coordinates to both explore the data and to visualize the relationships between the taxa in morphospace. Data were plotted using dietary categories to assist in the identification of patterns among observations, as well as by clade. Plots of the two clades were fitted with 95% confidence ellipses to further illuminate group‐based clusters. PC scores from the first and second components of each PCA (by tooth position) were plotted to enable comparisons to phylogenetic‐based methods (see below), where the first component is representative of data in which phylogenetic signal has either been minimized or maximized by redistributing the signal across higher PC axes (Revell 2009; Collyer and Adams 2021).

Two additional PCAs were implemented using the genus average of the Procrustes coordinates to examine how dietary and phylogenetic signals are distributed within the data. The first, a phylogenetic PCA (pPCA) finds the greatest axis of variation which is largely independent of a phylogenetic signal without the removal of said signal (reviewed in Polly et al. 2013). The phylogenetic signal is retained along subsequent axes such that the first component is most informative in the examination of phylogenetically controlled shape variation (Revell 2009; Polly et al. 2013; Collyer and Adams 2021). The second, a phylogenetically aligned PCA (PACA) seeks to align the data *to* the phylogenetic signal such that the first component is representative of the greatest proportion of covariance between the data and the phylogeny; and this is most informative for examining phylogenetic influence on shape variation (Collyer and Adams 2021). Because these methods explore the data in opposing ways (i.e., minimizing, or maximizing phylogenetic signal) they are complimentary analyses (Collyer and Adams 2021). All PCA analyses were performed using the function “gm.prcomp.” Plots as well as *K*_mult_ statistics for the first and second components of the pPCA and PACA were used to understand the performance of each analysis. Finally, because neither method controls for phylogenetic signal, data are highly correlated with one another and phylogenetic correction is required for subsequent analyses (Polly et al. 2013; Collyer and Adams 2021). To test the relationship between dietary signal and shape after controlling for evolutionary relationships, the first component of the pPCAs and PACAs were fitted to phylogenetic Procrustes ANOVAs (PGLS) using the function “procD.pgls.” Models used the grouping variable diet with 10,000 permutations.

## 3 Results

The Procrustes ANOVA for the combined tooth positions is statistically significant (*p <* 0.05). Pairwise comparisons show differences between the *M*_3_ and both *M*_1_ and *M*_2_ (*p <* 0.0001), whereas no significant difference is indicated between the *M*_1_ and the *M*_2_, though this comparison does approach significance (*p =* 0.05). Subsequently, downstream analyses focus on each tooth position independently to better understand their unique morphologies.

### 3.1 Allometry

Regressions of Procrustes coordinates for all tooth positions (*M*_1_, *M*_2_, and *M*_3_) on centroid size are statistically significant *p* < 0.05 but *r* ^2^ values for each model are low (*M*_1_ = 0.096, *M*_2_ = 0.064, *M*_3_ = 0.062) indicating very weak effects of allometry on the root cross‐sectional shape. Results of ANOVA RRPP models on the residuals for each tooth demonstrate statistically significant differences are present between all pairwise dietary comparisons at *M*_1_ except between mixed folivores and omnivores (Table 2). For the *M*_2_, mixed folivores do not differ from omnivores or soft object frugivores (Table 3). Statistically significant differences are found at the *M*_3_ for all dietary group comparisons except between mixed folivores and omnivores or hard object frugivores (Table 4).

**TABLE 2:** Pairwise distances between means for dietary categories using *M*_1_ residuals from the regression of centroid size on aligned coordinates (above diagonal) and associated *p*‐values (below the diagonal).

	Folivore	Mixed Folivore	Omnivore	Soft object Frugivore	Hard object Frugivore
Folivore	1.000	0.0657362	0.0600480	0.0929100	0.1164266
Mixed folivore	**0.0118**	1.000	0.0430581	0.0483243	0.0861769
Omnivore	**0.0243**	0.1895	1.000	0.0666044	0.0829249
Soft object frugivore	**< 0.001**	**0.0427**	**< 0.001**	1.000	0.0605831
Hard object frugivore	**< 0.001**	**< 0.001**	**< 0.001**	**0.001**	1.000

*Note:* Significant values are bolded (*p* < 0.05).

**TABLE 3:** Pairwise distances between means for dietary categories using *M*_2_ residuals from the regression of centroid size on aligned coordinates (above diagonal) and associated *p*‐values (below the diagonal).

	Folivore	Mixed Folivore	Omnivore	Soft object Frugivore	Hard object Frugivore
Folivore	1.000	0.0782463	0.0614710	0.0920409	0.1222309
Mixed folivore	**0.0031**	1.000	0.0478207	0.0388343	0.0818961
Omnivore	**0.0242**	0.1064	1.000	0.0637989	0.0893576
Soft object frugivore	**< 0.001**	0.3651	**0.0028**	1.000	0.0706598
Hard object frugivore	**< 0.001**	**< 0.001**	**< 0.001**	**< 0.001**	1.000

*Note:* Significant values are bolded (*p* < 0.05).

**TABLE 4:** Pairwise distances between means for dietary categories using *M*_3_ residuals from the regression of centroid size on aligned coordinates (above diagonal) and associated *p*‐values (below the diagonal).

	Folivore	Mixed Folivore	Omnivore	Soft object Frugivore	Hard object Frugivore
Folivore	1.000	0.1183604	0.1143881	0.1530413	0.1314517
Mixed folivore	**< 0.001**	1.000	0.0569454	0.0691288	0.0576839
Omnivore	**< 0.001**	0.1031	1.000	0.0860947	0.0682916
Soft object frugivore	**< 0.001**	**0.0142**	**< 0.001**	1.000	0.0566271
Hard object frugivore	**< 0.001**	0.1037	**0.0116**	**0.0294**	1.000

*Note:* Significant values are bolded (*p* < 0.05).

### 3.2 Phylogenetic Signal

Across the molar row, phylogenetic signal is consistently less than expected under the Brownian motion model for the Procrustes coordinate data as well as centroid size. *M*_1_ Procrustes coordinate results return a phylogenetic signal that is less than 1, but statistically significant (*K*_mult_ = 0.75, *p* < 0.05). The *M*_2_ Procrustes coordinates have less phylogenetic signal than the model expectation and that of *M*_1_ (*K*_mult_ = 0.69, *p* < 0.05). *M*_3_, phylogenetic signal is the same as *M*_1_ for the Procrustes coordinates (*K*_mult_ = 0.75, *p* < 0.05). The phylogenetic signal for centroid size across all molars is not statistically significant.

### 3.3 Standard PCA

**FIGURE 3:**
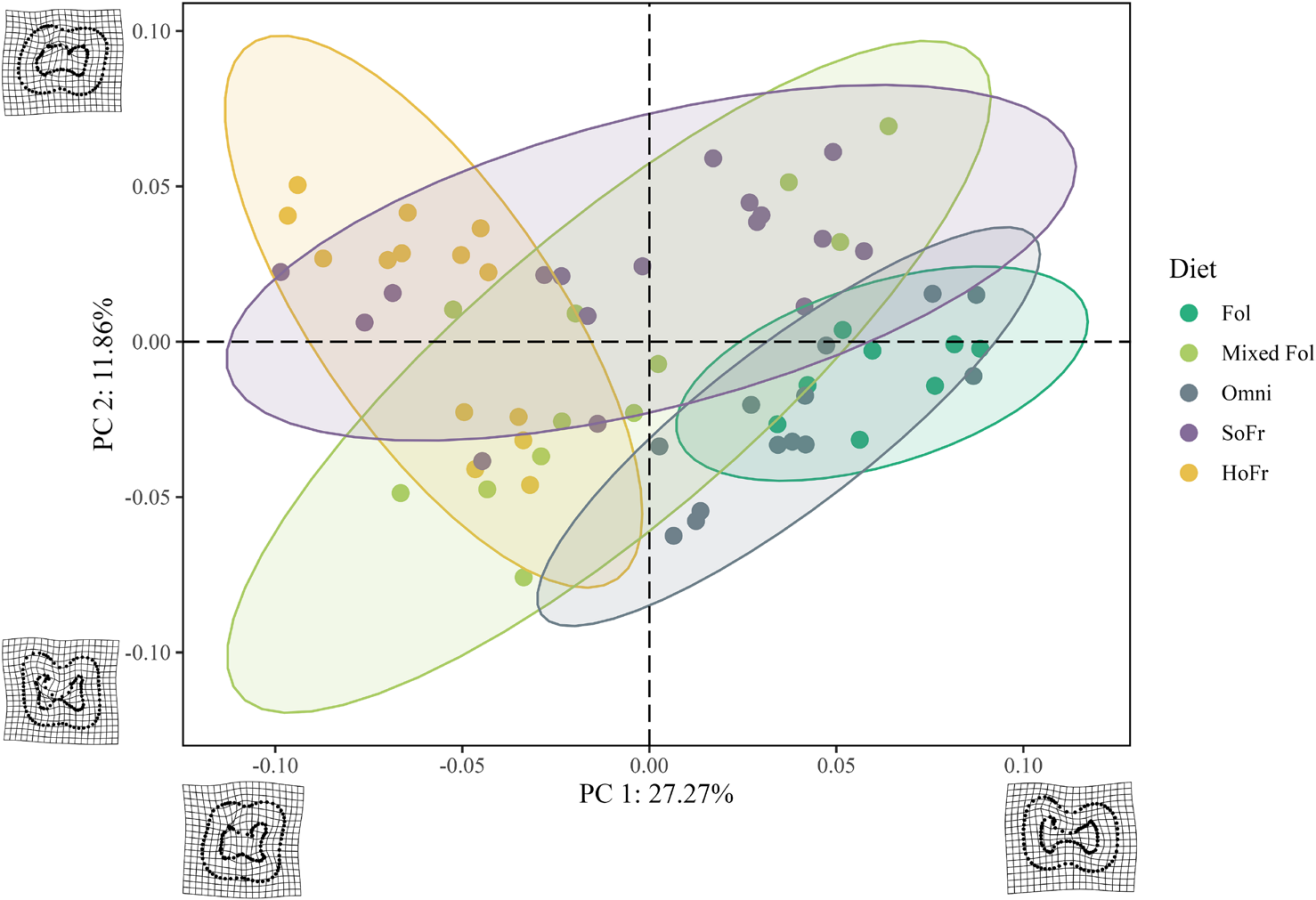
PCA bivariate plot of PC1 versus PC2 for M_1_. 95% confidence ellipses colored by diet. PCA deformation grids are along each axis. Mesial is left, buccal is toward the top of the figure.

**FIGURE 4:**
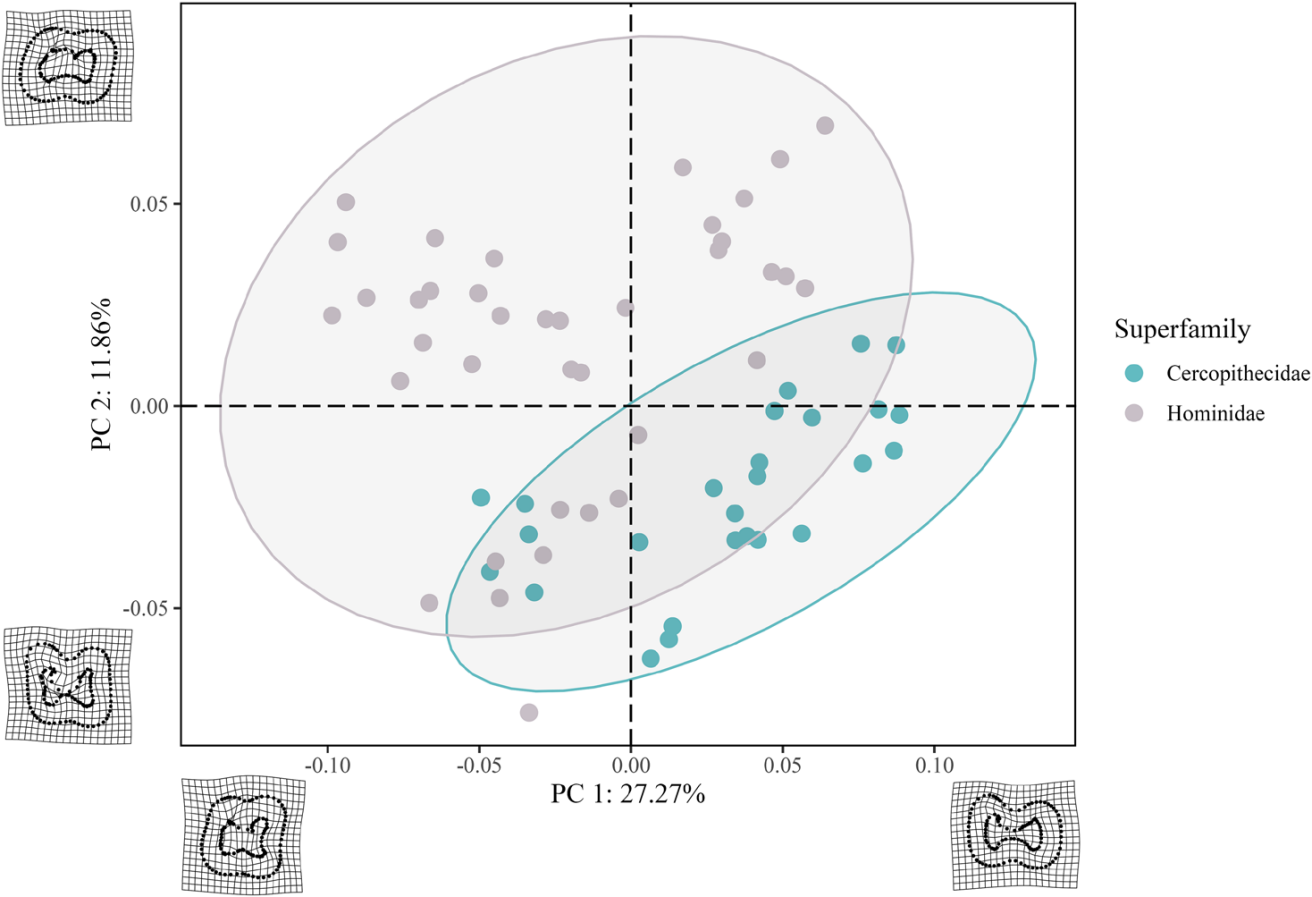
PCA bivariate plot of PC1 versus PC2 for *M*_1_. 95% confidence ellipses colored by superfamily. Deformation grids are along each axis. Mesial is left, buccal is toward the top of the figure.

The first and second principal components (PCs) for the *M*_1_ capture 39.13% (PC1 = 27.27; PC2 = 11.86) of the sample variation; with the first 20 PCs describing 89.9% of the sample variation. Across PC1, the negatively loaded hard object frugivores cluster away from the positively loaded omnivores and folivores (Figure 3). Shape changes along PC1 (Figure 3) are characterized by an increase in mesial and distal dentin as well as a narrowing of the pulp cavity moving from a more quadrate to an elongate molar. PC2 provides separation of many soft object frugivores with positive loadings from the predominately negatively loaded folivores. Shape changes across PC2 are dominated by the transition from an infilled pulp cavity with the presence of a strong buccal root groove to a reduction in the mesiobuccal dentin, a weakly developed root groove, and little pulp cavity infilling. The remaining groups are not well distinguished in morphospace. When plotted by clade and fitted with 95% confidence ellipses (Figure 4), hominoids and cercopithecoids form two clusters largely separated along PC2.

**FIGURE 5:**
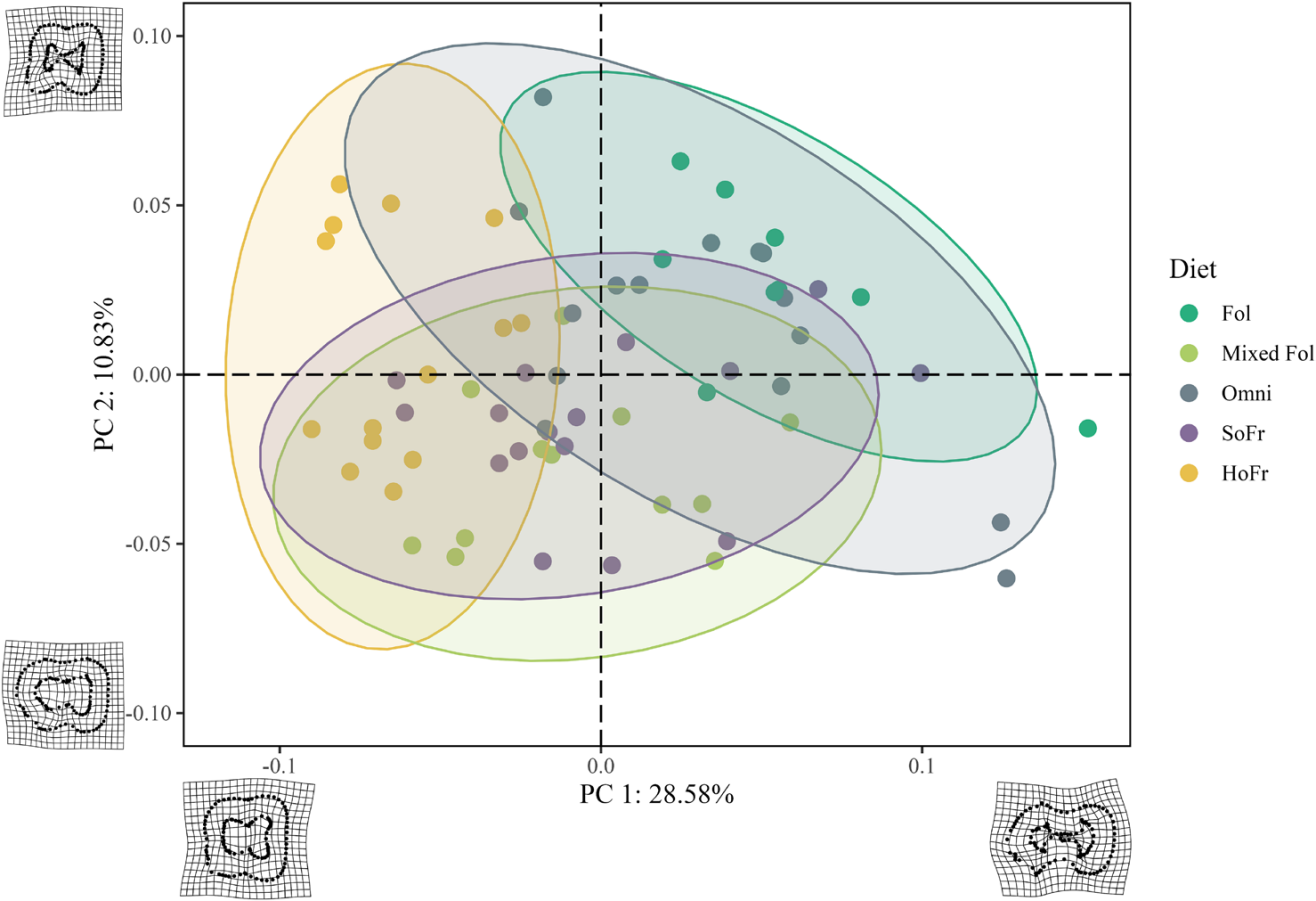
PCA bivariate plot of PC1 versus PC2 for *M*_2_. 95% confidence ellipses colored by diet. Deformation grids are along each axis. Mesial is left, buccal is toward the top of the figure.

**FIGURE 6:**
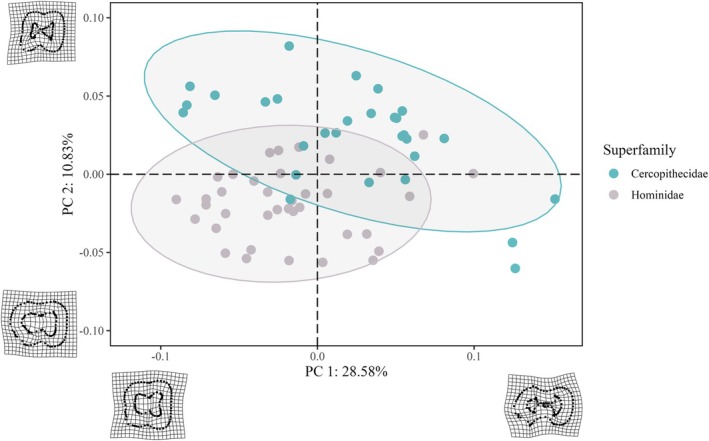
PCA bivariate plot of PC1 versus PC2 for *M*_2_. 95% confidence ellipses colored by superfamily. Deformation grids are along each axis. Mesial is left, buccal is toward the top of the figure.

A PCA of the *M*_2_ data describes 89.8% of the total variation in the first 20 components. The first two PCs capture 39.4% (PC1 = 28.58; PC2 = 10.83) of the sample variation. PC1 best divides hard object frugivores (negative PC space) from folivores (positive PC space). Omnivores, soft object frugivores, and mixed folivores largely cluster around the center of the morphospace (Figure 5). The shape changes at PC1 are driven by a large increase in buccal dentin, as well as a midline increase in mesial dentin. Bilateral infilling of the pulp cavity is accompanied by broad but weak, bilateral root grooves (Figure 5). For PC2, most soft object frugivores are separated from the folivores, with the folivores occupying a large portion of positive PC space. PC2 shape changes are characterized by a slight reduction in mesial dentin, a large increase in mesiobuccal dentin, and pulp cavity infilling. A clade‐based plot with 95% confidence ellipses provides separation of hominoids from cercopithecoids along PC2 (Figure 6).

**FIGURE 7:**
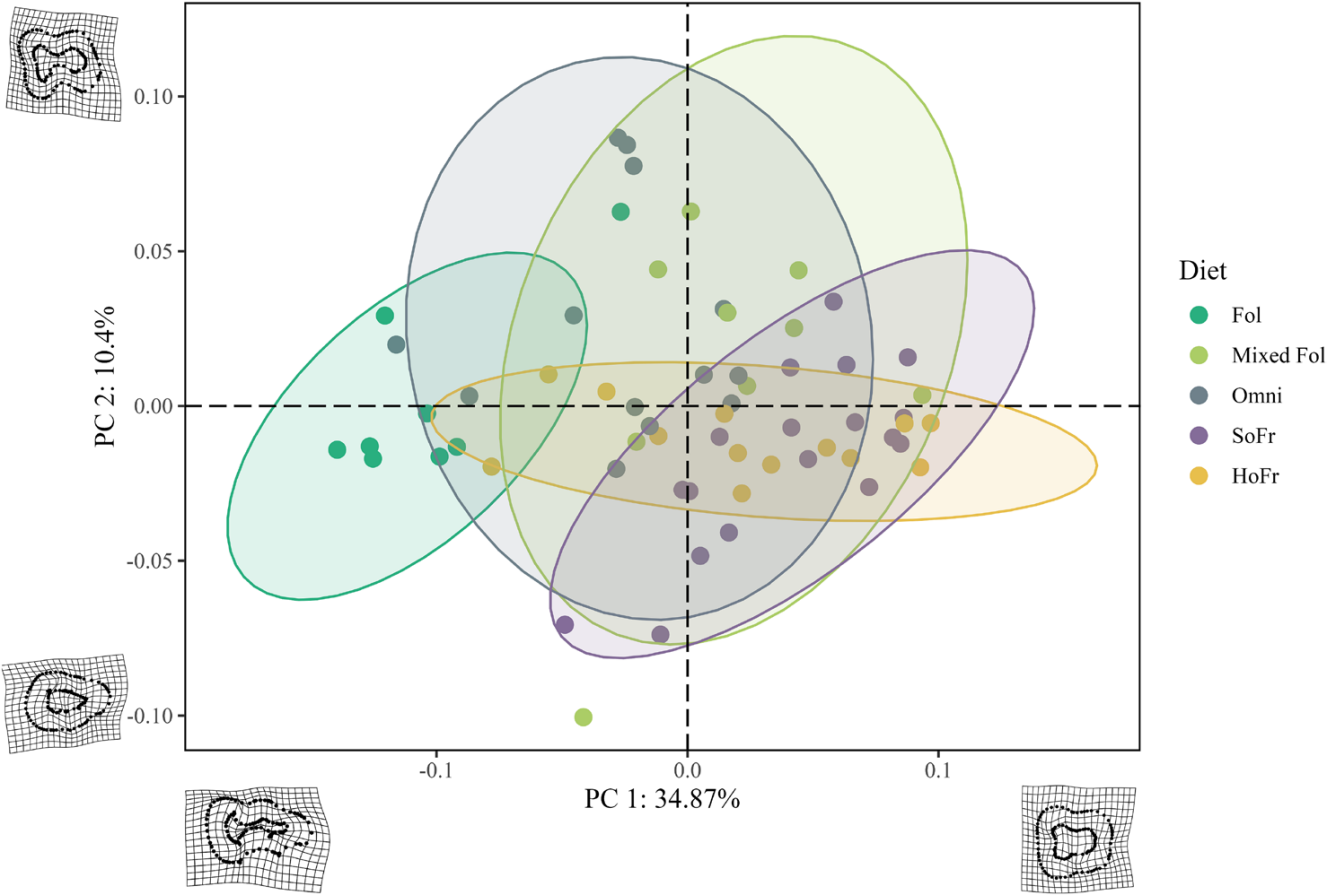
PCA bivariate plot of PC1 versus PC2 for *M*_3_. 95% confidence ellipses colored by diet. Deformation grids are along each axis. Mesial is left, buccal is toward the top of the figure.

**FIGURE 8:**
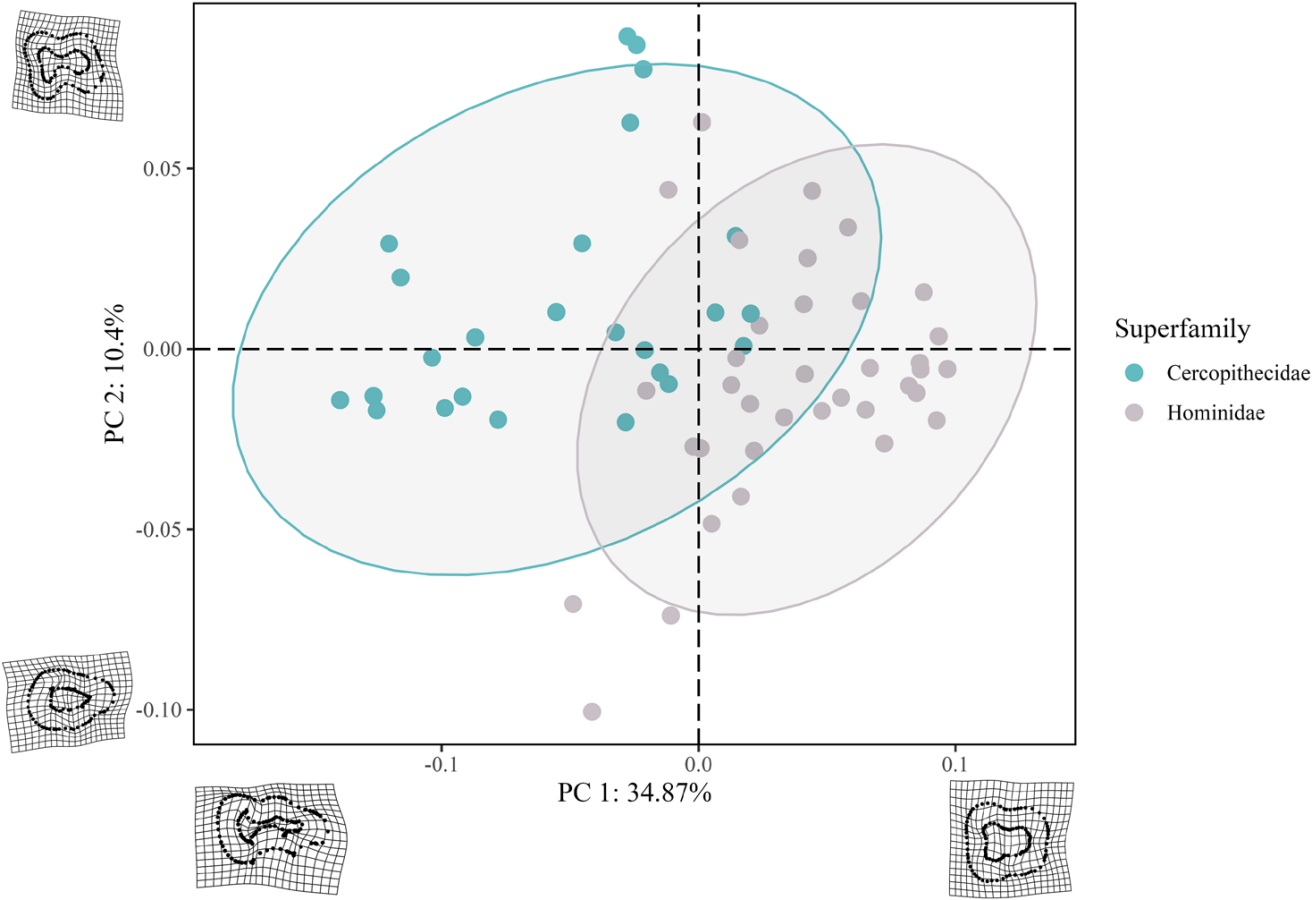
PCA bivariate plot of PC1 versus PC2 for *M*_3_. 95% confidence ellipses colored by superfamily. Deformation grids are along each axis. Mesial is left, buccal is toward the top of the figure.

Together the first two components for the *M*_3_ PCA explain 45.27% of the variation in the sample (PC1 = 34.87; PC2 = 10.4). Combined, the first 18 PCs describe 90% of the variation in this sample (Figure 7). Morphospace for PC1 generally provides group separation of negatively loaded folivores from the more positively loaded soft object frugivores. Changes in shape occurring across PC1 are largely driven by the reduction in buccal dentin and a broadening of the buccal lingual dimensions with a concomitant decrease in the pulp cavity (Figure 7). In contrast to the *M*_1_ and *M*_2_ tooth positions, along PC2, there is very little distinction between dietary groups. Along PC2 shape change is moderate with a reduction in the distal dentin accompanied by slight distobuccal and distolingual broadening. There is some pulp cavity infilling and the development of a lingual root groove. In a clade‐based plot with 95% confidence ellipses the two clades overlap significantly along the two axes, and neither group is well defined (Figure 8).

### 3.4 pPCA and PACA

**FIGURE 9:**
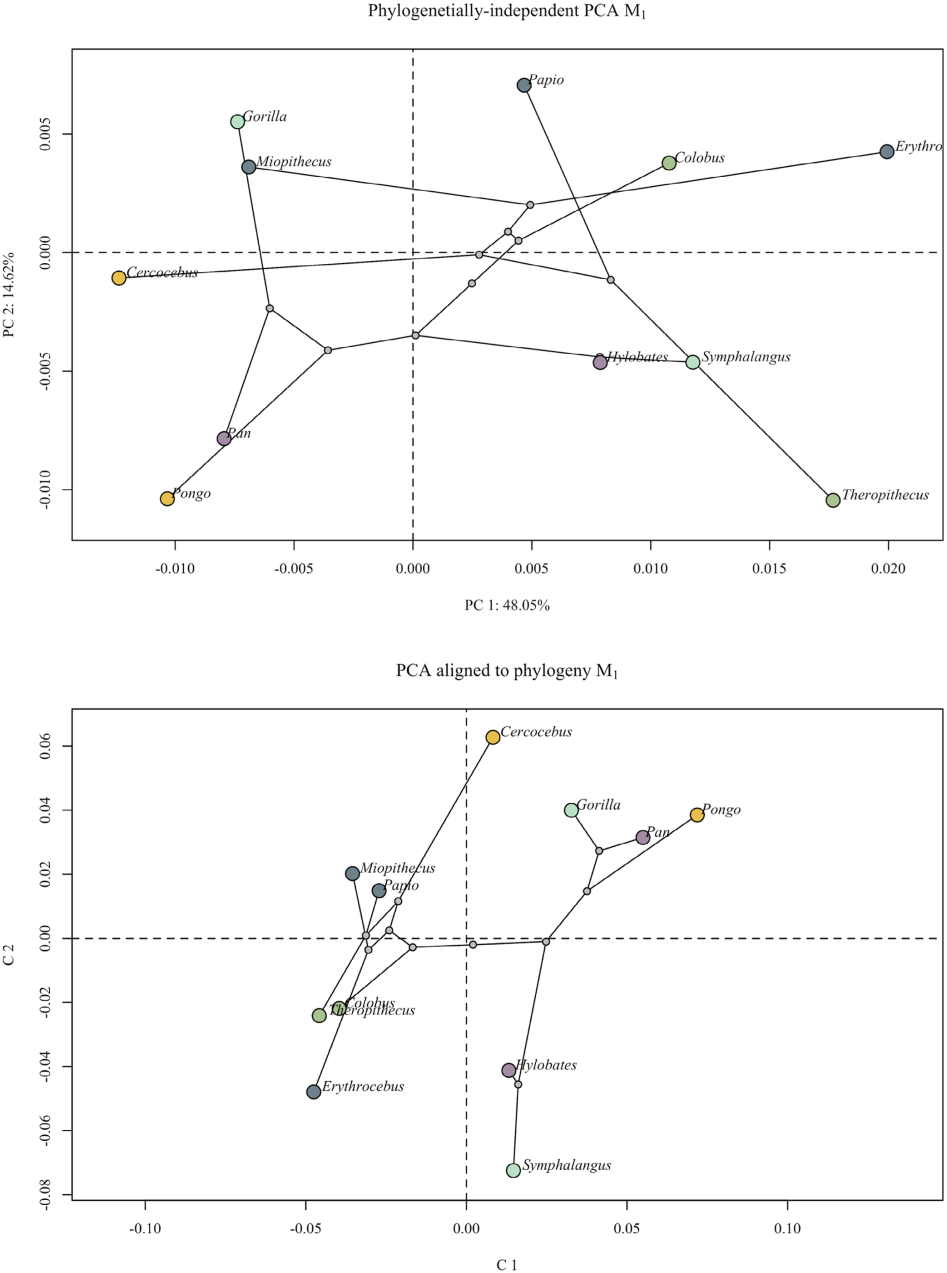
Comparison of phylomorphospace for the pPCA (top) and PACA (bottom) of *M*_1_. Genus average colored by diet.

Owing to the weak influence of size on shape, and because the phylogenetic signal for centroid size is less than would be expected under the Brownian motion model for all tooth positions, the following analyses were performed on the uncorrected Procrustes coordinates. The pPCA for the *M*_1_ position summarizes 62.67% of the shape variation in the first two components (PC1 = 48.05; PC2 = 14.62) (Figure 9, top). The phylogenetic signal for PC1 is not statistically significant and is lower than the original signal found in the Procrustes coordinates (*K*_mult_ = 0.63, *p =* 0.31), as is PC2 (*K*_mult_ = 0.41, *p =* 0.87). The proportion of covariance between the phylogeny and the data for the first two components of the *M*_1_ PACA is highest for component 1 (C1) at 28.38% and 3.38 for component 2 (31.76% combined) (Figure 9, bottom). The phylogenetic signal for the first component is stronger than the signal found in the Procrustes coordinates and statistically significant (*K*_mult_ = 1.62, *p <* 0.05). For component 2 (C2), the phylogenetic signal falls substantially closer to the level observed in the Procrustes coordinates but without statistical significance (*K*_mult_ = 0.74, *p =* 0.16).

The results from the pPCA phylogenetic Procrustes ANOVAs (PGLS) for PC1 are not significant between dietary categories (*p =* 0.27). *M*_1_ PACA first component (C1) results also lack statistical significance (*p =* 0.07). As such, pairwise comparisons were not performed.

**FIGURE 10:**
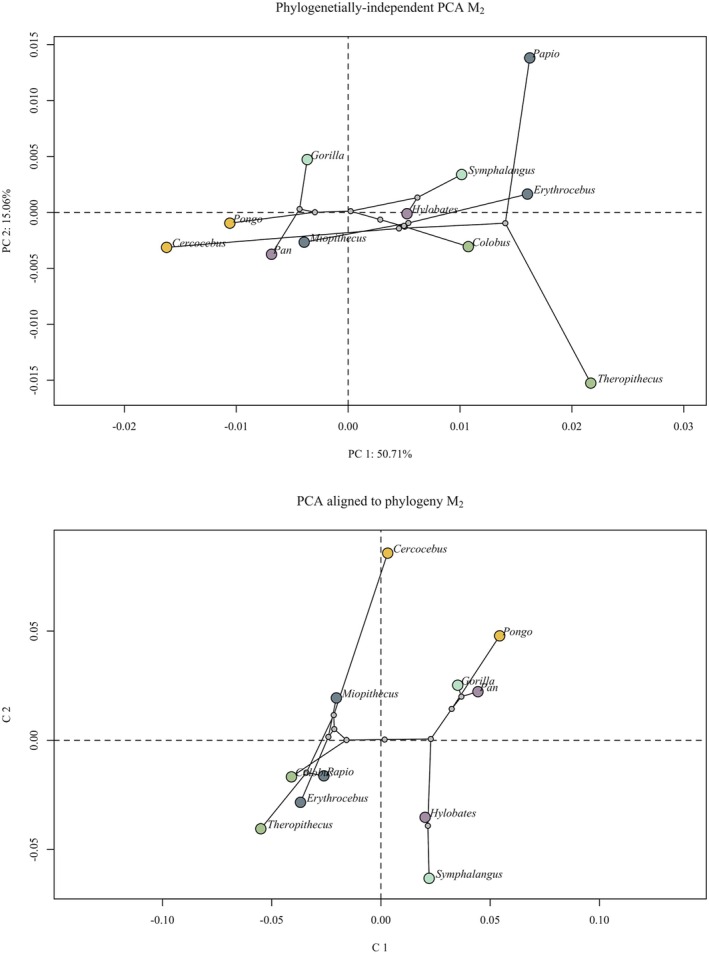
Comparison of phylomorphospace for the pPCA (top) and PACA (bottom) of *M*_2_. Genus average colored by diet.

At *M*_2_, the pPCA accounts for 65.77% of the sample variance in the first two components (PC1 = 50.71; PC2 = 15.06) (Figure 10, top). For PC1 and PC2, phylogenetic signal is not statistically significant. At PC1, the value of *K*_mult_ is close to the original value found in the Procrustes coordinates but without statistical significance (*K*_mult_ = 0.66, *p =* 0.25); while PC2 drops substantially and *K*_mult_ = 0.26 (*p =* 0.99). *M*_2_ PACA covariance between the phylogeny and the data partitions 28.5% across the first two components (C1 = 25.8; C2 = 2.7) (Figure 10, bottom). The phylogenetic signal of C1 is *K*_mult_ = 1.64 (*p <* 0.05) stronger than the signal found in the Procrustes coordinates (where *K*_mult_ = 0.69, *p* < 0.05). The C2 phylogenetic signal is lower than the level observed in the Procrustes coordinates but is not statistically significant (*K*_mult_ = 0.67, *p =* 0.24).

*M*_2_ pPCA PGLS for PC1 is not statistically significant (*p =* 0.09) between dietary categories. The PACA C1 results are significant (*p <* 0.05). The group mean distances (Table 5) are statistically significant for comparisons between folivores and mixed folivores (*p =* 0.05), hard object frugivores (*p <* 0.001), and soft object frugivores (*p =* 0.04).

**TABLE 5:** *M*_2_ PGLS of the first PACA component (C1).

	Folivore	Mixed Folivore	Omnivore	Soft object Frugivore	Hard object Frugivore
Folivore	1.000	0.0765786	0.0201962	0.0802955	0.0767033
Mixed folivore	**0.03315**	1.000	0.0563824	0.0037169	0.0001246
Omnivore	0.58380	0.11500	1.000	0.0600993	0.0565070
Soft object frugivore	**0.02135**	0.92480	0.08535	1.000	0.0035923
Hard object frugivore	**0.03145**	0.99920	0.10860	0.92430	1.000

*Note:* Pairwise distances between means for dietary categories using *M*_2_ (above diagonal) and associated *p*‐values (below the diagonal). Significant values are bolded (*p* < 0.05).

**FIGURE 11:**
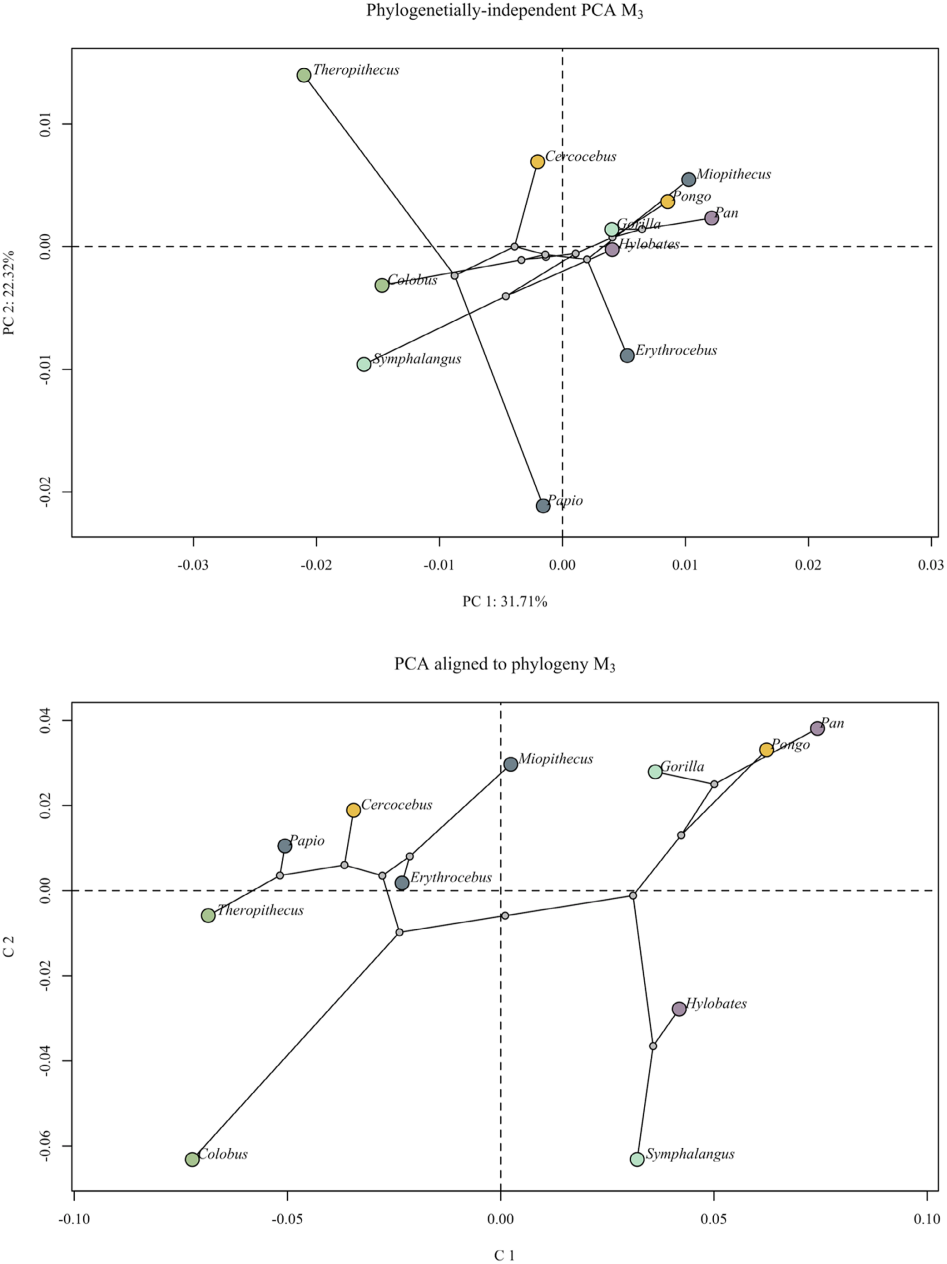
Comparison of phylomorphospace for the pPCA (top) and PACA (bottom) of *M*_3_. Genus average colored by diet.

The first two PCs from the *M*_3_ pPCA describe 54.03% of the variation in the sample (PC1 = 31.71; PC2 = 22.32) (Figure 11, top). The *K*_mult_ value representing the strength of the phylogenetic signal is lower than that observed for the Procrustes coordinate data at the *M*_3_ and is not statistically significant (*K*_mult_ = 0.53, *p =* 0.51). Along PC2, the same pattern observed for the *M*_1_ and the *M*_2_ is present with the signal dropping below the *K*_mult_ value of the first component (*K*_mult_ = 0.29, *p =* 0.99). The first two components of the *M*_3_ PACA (Figure 11, bottom) account for 42.4% of the covariance between the data and the phylogeny (C1 = 40.0; C2 = 2.4). Values of *K*_mult_ for C1 and C2 are both stronger than that of the Procrustes coordinates and both measures are statistically significant (*K*_mult_ = 1.90, *p <* 0.05 and *K*_mult_ = 0.99, *p <* 0.05, respectively).

In an interesting reversal from the other tooth positions, the PGLS results for the *M*_3_ pPCA are statistically significant (*p <* 0.05). Pairwise comparisons (Table 6) of group mean distances are significant between folivores and omnivores (*p =* 0.01).

**TABLE 6:** *M*_3_ PGLS of the first pPCA component (PC1).

	Folivore	Mixed folivore	Omnivore	Soft object frugivore	Hard object frugivore
Folivore	1.000	0.0069447	0.0212347	0.0219106	0.0178172
Mixed folivore	0.6903	1.000	0.0142900	0.0149659	0.0108725
Omnivore	**0.0148**	0.3962	1.000	0.0006759	0.0034175
Soft object frugivore	0.1869	0.0791	0.9677	1.000	0.0040934
Hard object frugivore	0.1522	0.4498	0.7876	0.7833	1.000

*Note:* Pairwise distances between means for dietary categories using *M*_3_ (above diagonal) and associated *p*‐values (below the diagonal). Significant values are bolded (*p* < 0.05).

The *M*_3_ PACA C1 does not achieve statistical significance (*p =* 0.3) for the comparison between dietary categories.

## 4 Discussion

The goal of this study was to understand the relationship between signals of dietary ecology (dietary categories), phylogenetic relationships, and cervical root morphology where both shape and size were considered. To accomplish this, I used 2D landmark data summarized by a variety of PCAs, and successive statistical evaluation of the resulting components, to explore whether patterns of root morphology in extant catarrhines are informative of diet or shared evolutionary history. Very weak allometric signals were detected at each tooth position and an abundant dietary signal was present when size‐corrected residuals were tested. As a result of the lack of allometric influence, subsequent analyses used uncorrected Procrustes coordinates. While the measurements of phylogenetic signal were less than expected under a Brownian motion model of evolution, PCA plots demonstrated that a clear phylogenetic structure was present within the data for each tooth position. Further analysis via phylogenetically independent and phylogenetically aligned PCA methods showed that signals of dietary ecology are closely intertwined with phylogenetic signals and redistribution of the phylogenetic signal across components also redistributes the dietary signal in phylomorphospace dampening the ability to detect dietary ecology profiles in shape space.

### 4.1 Allometry

Results for the analysis of centroid size and root morphology are interesting because while each tooth position demonstrated some signal, albeit very weak, the strength of this relationship decreased across the toothrow. There is a marginally stronger association between size and shape for the *M*_1_ (*r* ^2^ = 0.096), than for the other two teeth (*M*_2_ = 0.064 and *M*_3_ = 0.062); yet analysis of the residuals still indicated significant mean differences among most of the dietary categories across all tooth positions with the consistent exception of mixed folivores and omnivores. The need to process a variety of food items, some of which are overlapping both categorically and in food material properties like bark, grasses, seeds, and leaves, may impact loading patterns and ultimately produce convergent morphological patterns (Remis 1997; Hill and Dunbar 2002; Enstam and Isbell 2007; Hohmann 2009). The similarity in morphology demonstrated by mixed folivores and omnivores may be indicative of selective pressures to support the crown during the repeated processing of mechanically challenging fallback foods, as has been suggested to influence the crown in great apes (e.g., Constantino et al. 2009). If there is strong selective pressure to process mechanically challenging foods, then there should be a degree of overlap between the catarrhines in this study and more distantly related platyrrhine species that share this dietary adaptation such as *Alouatta* which is known to process stems and woody plant material (Anapol and Lee 1994; Dunn et al. 2012).

A weak allometric relationship between size and tooth roots has previously been found in *Gorilla* and *Pan* by Kupczik et al. (2018) with their 3D landmark analysis of root morphology. For these two taxa, the effects of allometry were similarly weak (*r* ^2^ = 0.07), but groups (defined by species) remained statistically different (Kupczik et al. 2018). When the dentition is being analyzed, allometry seems to hold some degree of influence on shape (e.g., Singleton et al. 2011; Gamarra et al. 2016; Kupczik et al. 2018). Taking allometry into account can impact the ability to detect other signals like phylogeny (e.g., Gamarra et al. 2016); however, accounting for allometry offered no meaningful benefit for this sample. Unlike previous research this study used 2D data from cross‐sections, but despite this methodological difference, the allometric signal was consistent with published data on large‐bodied hominoid dental roots (Kupczik et al. 2018) and cercopithecoid dental crowns (Gamarra et al. 2016). The results of the current study suggest that phylogeny and dietary ecology, rather than allometry, are driving the morphological variation demonstrated by the root cervix.

### 4.2 Phylogenetic Signal

Shape data for the root cervix were found to have significant measures of phylogenetic signal for each tooth position, though all values were less than expected under Brownian motion. This result is mirrored in PCA plots coded by clade of the Procrustes coordinates which demonstrate phylogenetic structure within the data with hominoids and cercopithecoids showing differences in both variation and directionality with some overlap (Figures 4, 6, and 8). The phylogenetic signal for centroid size was not significant for any tooth position and consistent with the allometry results, size is not likely a major contributor to phylogenetic structure in the data. This result may reflect the analysis, that is, the cervical root cross‐section contains information about dentin thickness and shape and does not retain any volumetric information. Measures of phylogenetic signal for the molar crown are consistent with the results presented in this study, though the methods differ. For example, Gamarra et al. (2016) used twelve 2D landmarks on the first and second lower molars of over 750 extant and fossil primates to understand the influence of phylogeny on molar morphology. Using Mantel's test to compare morphologic and genetic distance matrices, the authors found that the phylogenetic signal was strongest at the superfamily level and less strong, but still significant when the groupings were family level or lower (Gamarra et al. 2016). The data in this study were measured using genus‐level groupings and, as such, the strength of the signal is on par with that of the crown.

### 4.3 PCA Morphospace

For all three molars, shape changes along PC1 are largely driven by reductions in the dimensions of the pulp cavity space (Figures 3, 5, and 7). This tends to separate hard object frugivores from other groups and may indicate that additional dentin is reinforcing the cervix for processing mechanically challenging food objects like seeds or unripe fruits in a way that is distinct from the processing of other food items or during repetitive loading as in folivores. This aligns with previous results from Sims (2025) where hard object frugivores demonstrated higher values of dentin area relative to soft object frugivores and provides additional evidence that changes in the pulp cavity dimensions may be driving the difference observed in dentin area. The PCA results are also consistent with the study by Selig et al. (2021) which examined pulp chamber volumes in the primates *Pongo*, *Pan*, *Sapajus*, *Cebus*, *Pithecia*, and *Plecturocebus* which were categorized as either having low or high wear diets. High‐wear species (*Pongo*, *Sapajus*, and *Pithecia*) had more pulp as a percentage of tooth volume, and dentin deposition on areas of wear (facets) was observed in the sample. It is possible that folivores do not require as much pulp volume as seed predators owing to less fracture risk from leaf and stem consumption (Constantino et al. 2009), though the incidence of dentin fracture remains understudied. Interestingly, the folivores demonstrate pulp cavity constriction that, unlike hard object frugivores, is the result of consistent midline bilateral deposits across all three molars. This result is similar to the pattern identified by Sims (2025) in that the primates that engaged in repetitive loading (and forceful biting) had buccal‐lingually oriented dentin distribution patterns. Unlike the cross‐sectional geometry results, however, more nuanced regions are identifiable with the landmark‐based analysis.

Observed variation across all tooth positions in the relative dimensions of the pulp cavity indicate a degree of phenotypic plasticity in the cervical root cross‐section that appears to be driven by as yet unidentified aspects of dietary ecology, one potential driver are the material properties of habitually consumed items. The molar cervical morphology for this sample may reflect patterns of adult dentin deposition in response to wear that is specific to the material properties of the foods being consumed (e.g., Galbany et al. 2014). A functional link between food material properties on the mandible has been documented in both *Gorilla* and *Pongo* (Taylor 2002, 2006), and in the topology of the upper molars of Papionins (Avià et al. 2022). The relationship between measures of toughness, the resistance to elastic deformation (measured using Young's modulus), and dental morphology, however, is complex (Coiner‐Collier et al. 2016). There is not yet a clear understanding of how the odontoblast population in the pulp cavity responds to diets with varying mechanical properties which may change how we view molar cervical morphology if indeed there is a form‐function link.

PCA plots that include clade‐based confidence ellipses show that the influence of phylogeny is present along PC2 for both *M*_1_ and *M*_2_ (Figures 4 and 6). Across this axis, clustering patterns for both diet and superfamilies can be visualized, suggesting that these data hold both dietary and phylogenetic signals. Interestingly, the most distal tooth position, the *M*_3_, lacks the phylogenetic structure observed for the other two teeth (Figure 8). This may be due to a high degree of morphological variation in the *M*_3_ that has been previously noted in an examination of fossil hominin roots (Wood et al. 1988), and suggested to be an effect of gene inhibition on molar size (Kavanagh et al. 2007).

Consideration of the potential effects of developmental processes may help to illuminate the observed variation in the morphology of the *M*_3_. For example, the inhibitory cascade model suggests that the effects of gene activation and inhibition can lead to predictable patterns of tooth size across the tooth row (Kavanagh et al. 2007). However, this model is differentially supported across primate clades, with colobines and papionins following the model while cercopithecines and hominoids do not (Carter and Worthington 2016). Expansion of this sample to include non‐catarrhine primates may help to clarify how phylogenetic structure and developmental processes are impacting shape variation in primate *M*_3_s.

### 4.4 pPCA and PACA

The phylogenetically independent PCA (pPCA) was effective at redistributing the phylogenetic signal for all three tooth positions leading to a diminished phylogenetic signal with no statistical significance; however, this seemed to also redistribute the dietary signal, and diet could not be distinguished using the phylogenetic Procrustes ANOVAs except at the *M*_3_. Given that the standard PCA for the *M*_3_ lacked visible evidence of phylogenetic structure and all primates within the folivore and omnivore dietary categories are closely related cercopithecoids, dietary signals are likely a strong driver of morphological variation in the *M*_3_s of this clade. The phylogenetically aligned PCAs were also successful at realignment of the phylogenetic signal and increased signal measures with statistical significance were seen for all tooth positions. At *M*_2_ (but not *M*_1_ or *M*_3_) signals for diet could effectively be discerned with the PGLS. While this appears to indicate that the *M*_2_ position is best suited for this analysis, unequal sample sizes both in tooth position and across dietary categories (Table 1) may be impacting the statistical power of the analyses at *M*_1_ and *M*_3_. Combined these results are interpreted as indicating a strong link between dietary ecology and phylogeny in these data. Root morphology has previously been demonstrated to carry both phylogenetic and dietary signals yet the relationship between both variables as well as their influence on trends in shape is not well understood (Emonet et al. 2012). It seems reasonable that in most cases closely related organisms sharing phenotypic traits may also exploit similar or overlapping dietary niches. Of note is the placement of *Cercocebus* near *Pongo* across tooth positions in the phylomorphospace of both pPCA and PACA plots (Figures 9, 10, 11). Despite the evolutionary distance between them, these two taxa share a dietary strategy as hard object frugivores that seems to override the phylogenetic signal.

## 5 Conclusion

This work has shown that there are differences in the patterns of dentin distribution around the pulp cavity at the cervical margin and by focusing on a cross‐section that combines data from the external and internal regions important information about primate dietary ecology was uncovered. Across extant catarrhines, these shape differences have a weak allometric component that does not significantly influence the detection of dietary signals in these data. Overall, the morphology of the cervical root cross‐section provides an effective locale for assessing dietary ecology for most groups except mixed folivores and omnivores. Their shared morphology across tooth positions may represent an adaptation to processing foods with similar material properties. Thus, despite occupying distinct dietary niches, an overlap in processing behaviors and/or the food items themselves may be driving the observed similarities. To explicate a possible form‐function relationship, future research will incorporate animals from captive environments for which diet composition is known and material properties can be tested.

The morphology of the root cervix was found to be significantly influenced by phylogeny which is closely linked in these data to dietary ecology. As phylogenetic signal is redistributed across PC axes, the dietary signal may be carried with it; this has the effect of either weakening or enhancing its detection. The use of pPCA and PACA together helps to illuminate the distribution of these signals across morphospace. These analyses have also highlighted the need for teasing apart the components of the dietary ecology and phylogenetic signal in dental roots; this work will require the addition of more taxa and should focus on the use of a primate dataset that includes strepsirrhines and platyrrhines to sample the primate phylogeny more fully and to expand the range of dietary ecologies. Future research will examine how dietary and phylogenetic signals are distributed in the anterior dentition including premolars, which are often used in pre‐oral processing.

## Author Contributions

**Zana R. Sims:** conceptualization, investigation, funding acquisition, writing – original draft, methodology, writing – review and editing, formal analysis.

## Funding

This work was supported by the Leakey Foundation.

## Conflicts of Interest

The author declares no conflicts of interest.

## Data Availability

The data that support the findings of this study were previously published in Sims (2025) and available in the Supplement for that article. Original scans were collected using specimens on loan from the Harvard Museum of Comparative Zoology and micro‐CT scanned at the Harvard Center for Nanoscale Systems with support from a grant from the Leakey Foundation and are available on MorphSource.org under the project Sims MCZ Mandible Scans.
